# ALOX5AP Predicts Poor Prognosis by Enhancing M2 Macrophages Polarization and Immunosuppression in Serous Ovarian Cancer Microenvironment

**DOI:** 10.3389/fonc.2021.675104

**Published:** 2021-05-19

**Authors:** Xiang Ye, Limei An, Xiangxiang Wang, Chenyi Zhang, Wenqian Huang, Chenggong Sun, Rongrong Li, Hanlin Ma, Hongyan Wang, Min Gao

**Affiliations:** ^1^Department of Geriatric Medicine, Qilu Hospital of Shandong University, Jinan, China; ^2^Key Laboratory of Experimental Teratology of Ministry of Education, Department of Medical Genetics, School of Basic Medical Sciences, Cheeloo College of Medicine, Shandong University, Jinan, China; ^3^Health Management Division, Rizhao Central Hospital, Rizhao, China; ^4^Department of Obstetrics and Gynecology, Central Hospital Affiliated to Shandong First Medical University, Jinan, China; ^5^Department of Obstetrics and Gynecology, Gynecology Oncology Key Laboratory, Qilu Hospital of Shandong University, Jinan, China

**Keywords:** targeted therapy, serous ovarian cancer, ALOX5AP, immunosuppression, prognosis

## Abstract

**Background:**

Serous ovarian cancer (SOC) is a highly lethal gynecological malignancy with poor prognosis. Given the importance of the immune-related tumor microenvironment (TME) in ovarian cancer, investigating tumor-immune interactions and identifying novel prognostic and therapeutic targets in SOC is a promising avenue of research. ALOX5AP (Arachidonate 5-Lipoxygenase Activating Protein) is a key enzyme in converting arachidonic acid to leukotriene: a crucial immune-modulating lipid mediator. However, the role of ALOX5AP in SOC has yet to be studied.

**Methods:**

ALOX5AP expression patterns across ovarian cancer and their normal tissue counterparts were cross-checked using public microarray and RNA-seq analyses and then validated in clinical samples by qRT-PCR. Kaplan-Meier survival analysis was performed in multiple independent SOC patient cohorts. Univariate and multivariate Cox regression analysis were then employed to identify clinical risk parameters associated with survival, and a genomic-clinicopathologic nomogram was built. Gene enrichment, immune infiltration, and immunosuppressor correlation analyses were then evaluated.

**Results:**

ALOX5AP mRNA levels in SOC tissues were significantly upregulated compared to normal tissues. Elevated ALOX5AP was markedly associated with poor overall survival and progression-free survival in multiple SOC patient cohorts as well as with adverse clinicopathological features, including lymphatic invasion, unsatisfactory cytoreductive surgery, rapid relapse after primary treatment, and platinum non-responsiveness. A predictive nomogram, which integrated ALOX5AP expression and two independent prognosis factors (primary therapy outcome and tumor residual), was conducted to predict the 3-year and 5-year survival rate of SOC patients. Mechanistically, functional and pathway enrichment analyses revealed that ALOX5AP was primarily involved in immune response and regulation. Further exploration demonstrated that ALOX5AP was highly expressed in the immunoreactive subtype of ovarian cancer and closely related to immunocyte infiltration, especially M2 macrophage polarization. Additionally, ALOX5AP was enriched in the C4 (lymphocyte depleted) immune subtype of SOC and associated with crucial immune-repressive receptors in the tumor microenvironment at the genomic level.

**Conclusions:**

ALOX5AP expression indicates a worse survival outcome and has the potential to be utilized as a prognostic predictor for SOC patients. Given the availability of well-studied ALOX5AP inhibitors, this study has immediate clinical implications for the exploitation of ALOX5AP as an immunotherapeutic target in SOC.

## Introduction

Ovarian cancer is the number one cause of female reproductive related cancer deaths worldwide; in 2021, it is estimated that there will be 21,410 new cases diagnosed and 13,770 women will die of ovarian cancer in the United States alone (1). Ninety percent of ovarian cancers are epithelial, the most common (75%) being serous ovarian cancer (SOC) which is characterized by its aggressiveness, high recurrence rate and significant chemoresistance (2). Current standard therapy consists of a combination of debulking surgery and platinum-based chemotherapy (3). Unfortunately, despite therapy improvements, tiny residual lesions still exist after surgery and chemotherapy; 46% of patients with advanced stage of ovarian cancer will die within 5 years after diagnosis (4). There is an urgent need to explore novel therapeutic strategies to enhance SOC treatment efficiency. The past decade has seen a treatment revolution, moving away from drugs that target tumor cells toward modulating immune microenvironment against tumors, an approach orchestrated between various immunocytes and immune checkpoints (5). Patients who respond to immunotherapy achieve long-term benefits; successful treatment can entirely clear tumor lesions, preventing recurrence from tiny lesions as a result of immune surveillance and memory functions (6). Given the importance of the immune-related tumor microenvironment (TME) in ovarian cancers, investigating tumor-immune interactions and identifying novel prognostic and therapeutic targets in SOC has the potential to combat this deadly carcinoma.

Leukotrienes (LTs), derived from the nuclear membrane of cells, are crucial immunomodulating and proinflammatory lipid mediators, produced and excreted in response to various immune stimuli that have been implicated in several aspects of carcinogenesis: promoting chemotaxis and the activation of leukocytes (7). ALOX5AP (Arachidonate 5-Lipoxygenase Activating Protein) is a key enzyme needed for the production of leukotrienes through the 5-lipoxygenase (5-LOX) pathway (8). The human ALOX5AP is located on chromosome 13q12-13 and consists of 5 exons and 4 introns (9). Prior work pharmacologically targeting ALOX5AP points to an important role of the ALOX5AP/leukotriene pathway in adaptive immunological circuits of the inflammation response (10). Additionally, previous study has shown that ALOX5AP is universally expressed in 20 types of epithelial cancer cell lines. This conserved expression suggests that ALOX5AP may have a fundamental role in mediating cancer development (11). ALOX5AP has been reported to possess important prognostic significance in several cancer types, including colorectal cancer (12, 13), lung adenocarcinoma (14), low-grade glioma (15), and osteosarcoma (16–18). However, few studies on ALOX5AP and its potential role in ovarian cancer carcinogenesis have been reported.

Herein, using the valuable and reliable information provided by the open high-throughput transcriptomic database, we applied a series of bioinformatics algorithms to cross-check the expression and prognostic role of ALOX5AP across multiple independent ovarian cancer patient cohorts. We then systematically investigated the relation between ALOX5AP and clinicopathologic parameters, and developed a nomogram to predict the survival rate of SOC patients. Further mechanistic exploration revealed that ALOX5AP is strongly linked to immunocytes infiltration, particularly M2 macrophage abundance and immunosuppressor expression in the SOC microenvironment. This study indicated that ALOX5AP harbors great potential significance as a prognostic biomarker and an immunotherapy target for SOC.

## Materials and Methods

### Expression Analysis in Normal and Tumor Tissues

The TNMplot database (www.tnmplot.com) is a web tool for comparing gene expression in normal, tumor and metastatic tissues (19). It is the largest currently available transcriptomic cancer database consisting of 57,000 samples by utilizing multiple RNA-Seq and microarray datasets. TNMplot was employed to compare ALOX5AP mRNA expression in ovarian cancer and healthy controls based on either RNA-seq (TCGA and the Genotype-Tissue Expression project; GTEx) or gene array (NCBI-GEO) platforms. The online Gene Expression Profiling Interactive Analysis 2 (GEPIA2) database (http://gepia2.cancer-pku.cn) is a valuable web server for analyzing RNA-sequencing expression data of cancerous and healthy samples from the TCGA and the GTEx databases (20). ALOX5AP expression comparison was performed across 33 different cancer types using GEPIA2.

### Clinical Samples

All clinical samples, with written informed consent, were obtained from the Gynecology Department of Qilu Hospital of Shandong University. Patients were diagnosed with SOC and underwent initial debulking surgery, without prior chemotherapy or surgery. Fallopian tube (FT) tissues were obtained from patients who received salpingectomy due to benign gynecologic diseases. This study was approved by the Ethical Committee of Shandong University.

### RNA Isolation and qRT-PCR

Total RNA was isolated using TRIzol reagent (15596018, Invitrogen). In order to synthesize cDNA, PrimeScript RT Reagent Kit (RR037A, TaKaRa, Kyoto, Japan) was used. Gene expression was examined by quantitative real-time PCR (qRT-PCR) with SYBR Premix Ex Taq (RR420A, TaKaRa) and analyzed using a 7900HT Fast Real-Time PCR System (Applied Biosystems, Waltham, MA, USA). Details of quantitative PCR method were outlined in our previous report (21). Primers sequences for PCR reactions: ALOX5AP-F, CTGCGTTTGCTGGACTGATG; ALOX5AP-R, GGAGATGGTGGTGGAGATCG; GAPDH-F, CCACCCATGGCAAATTCCATGGCA; GAPDH-R, TCTAGACGGCAGGTCAGGTCCACC. mRNA levels of ALOX5AP were normalized against GAPDH using the double-delta cycle number of thresholds (ΔΔCt) method.

### Kaplan‐Meier Plotter Analysis

The Kaplan‐Meier Plotter database (www.kmplot.com) was used to assess the prognostic value of ALOX5AP in ovarian cancer patients (22). The high and low ALOX5AP expression groups were divided according to the auto select best cutoff-value of ALOX5AP. HR with 95% CI and p-values were calculated and extracted from the Kaplan‐Meier Plotter database and were shown in the plot.

### Predictive Value Analysis

Receiver operating curve (ROC) plotter (http://www.rocplot.org) is an online platform enabling the discovery, validation, and ranking of predictive treatment biomarker candidates in ovarian cancer (23). Treatment response was identified according to relapse-free survival at 12 months for serous ovarian cancer. ALOX5AP expression was compared between platinum responders and non-responders using the Mann–Whitney test. A ROC was drawn to analyze the predictive power of ALOX5AP in platinum responsiveness.

### Cancer Dependency Analysis

To explore the correlation between ALOX5AP and cisplatin sensitivity of ovarian adenocarcinoma cell lines, the Dependency Map (DepMap) database was utilized (https://depmap.org/portal/). The DepMap portal can systematically identify cancers genetic dependencies and small molecule sensitivities (24). Pearson’s correlation analysis was employed to evaluate statistical significance.

### Data Source and Identifying Differentially Expressed Genes (DEGs)

The RNA-sequencing data and corresponding clinical information for 489 SOC cases were downloaded from the Cancer Genome Atlas (TCGA) data portal (http://cancergenome.nih.gov/). Corresponding clinical data were obtained from Lui et al. (25). Level 3 HTSeq-FPKM files were downloaded and transformed into TPM (transcripts per million reads) for further analyses. After excluding samples without clinical details, 373 samples were retained for further analysis (**Supplement Table 1**). Expression values of ALOX5AP were dichotomized into low- and high-expression groups using the median as the cut-off value. Expression profiles between low and high ALOX5AP expression groups were compared to screen DEGs using an unpaired Student’s t-test *via* DESeq2 (version 3.10) software using R package edgeR (26). Genes with an absorbance fold change (logFC) > 1.5 and adjusted p-value < 0.05 were considered significantly DEGs. The results were visualized as volcano plots and heat map clusters drawn by R statistical 3.6.3 software.

### Functional Enrichment Analysis

Gene enrichment analyses for functional annotation into biological process categories of identified ALOX5AP related DEGs were performed using the web program Metascape (https://metascape.org) (27), using express analysis mode. Metascape provides a comprehensive gene annotation list based on multiple independent databases studying functional enrichment. Terms with a minimum count > 3, p-value < 0.01, and enrichment factor >1.5 (enrichment factor is the ratio between observed count and the count expected by chance) were collected and grouped into clusters based on their membership similarities.

### Pathway Enrichment Analysis

Gene Set Enrichment Analysis (GSEA) software was used to perform pathway enrichment analysis (28, 29). Statistical significance was determined using a 1000-fold permutation test. Gene set collections from Molecular Signatures Databases (MSigDB; https://www.gsea-msigdb.org/gsea/msigdb/index.jsp) were used in GSEA analysis. Enrichments were estimated using the absolute values of normalized enrichment score (NES) > 1. Significance of the enrichments was defined as an adjusted p-value < 0.05 and a false discovery rate (FDR) q-value < 0.25. R package clusterProfiler (version 3.10) was used to create the GSEA plot (30). Additionally, gene set variation analysis (GSVA), which is a GSE method to estimate pathway activity variation over a sample population in an unsupervised manner, was employed for further pathway analysis (31). This provides increased power to detect subtle pathway activity changes over a sample population in comparison to corresponding methods; p-value < 0.05 was used as the threshold. GSVA analysis was performed using R package GSVA (version 3.12).

### Immune Infiltration Analysis by ssGSEA

Immune signature score was calculated using single sample Gene Set Enrichment Analysis (ssGSEA2.0) implemented by a GSVA package in R (31). Based on the signature genes of 24 different immune cell types, relative immunocyte tumor infiltration levels were quantified from gene expression profiles for each tumor sample (32). The correlation between ALOX5AP and immune cell infiltration levels was analyzed by Spearman correlation, and the association of infiltration of immune cells with different ALOX5AP expression groups was analyzed by Wilcoxon rank sum test.

### TIMER 2.0 Analysis

Tumor IMmune Estimation Resource 2.0 (TIMER 2.0; http://timer.comp-genomics.org/) is a reliable tool that provides systematic evaluations of different immune cell infiltration (33). TIMER 2.0 was employed to investigate the relationship between ALOX5AP expression and the infiltration of different macrophage types in RNA‐seq human ovarian cancer samples from TCGA. The gene markers of M1 and M2 macrophages were referenced from prior studies (34).

### TISCH Analysis

Tumor Immune Single Cell Hub (TISCH; http://tisch.comp-genomics.org), is a large-scale curated database integrating single-cell transcriptomic profiles of ~2 million cells from 76 high-quality tumor datasets (35). TISCH was utilized to further verify ALOX5AP expression and immune cell infiltration in ovarian cancer at the single-cell level.

### TISIDB Analysis

The TISIDB database (http://cis.hku.hk/TISIDB) integrates multiple types of data resources in onco-immunology (36). Here, the TISIDB database was employed to cross-check ALOX5AP’s role in tumor-immune interactions through analyzing ALOX5AP expression in different cancer types as well as co-expression between ALOX5AP and immunosuppressor.

### Statistical Analysis

Statistical analysis was conducted using R statistical package (R version 3.6.2). Comparison between groups was evaluated *via* Wilcoxon rank sum test (unpaired). Relationships between clinical-pathologic features and ALOX5AP expression were compared using Wilcoxon signed-rank test, nonparametric Kruskal-Wallis, Wilcoxon Rank Sum test, or Spearman correlation. Univariate logistic regression analysis, Fisher exact test, and Pearson chi-square test were performed to search patients’ characteristics and differing ALOX5AP expression groups. Survival related analysis was conducted using univariate and multivariate Cox regression and the Kaplan-Meier method. After, a nomogram model and calibration plots were formulated based on the TCGA-OV cohort to predict 3‐ and 5‐year OS by including all independent prognostic factors using the rms package in R software. To further evaluate the discrimination ability of ALOX5AP in SOC, ROC curve analysis using pROC package was performed (37). A p-value < 0.05 was considered statistically significant in all tests.

## Results

### ALOX5AP Is Highly Expressed in SOC

**Figure 1:**
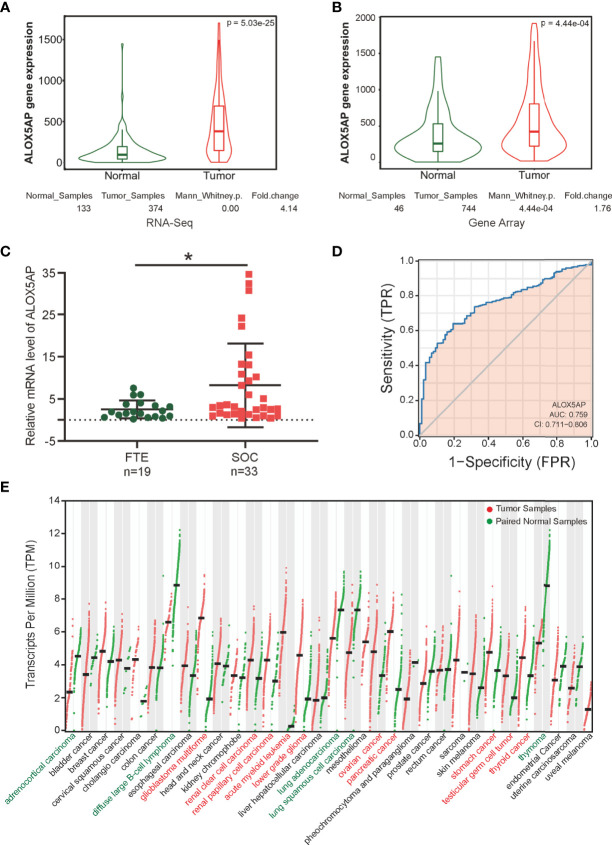
ALOX5AP expression in normal and cancer tissues. Comparison of ALOX5AP expression in SOC and normal controls based on **(A)** RNA-Seq analysis using TCGA-OV samples and matched normal control samples from the GTEx project, **(B)** microarray analysis using meta GEO ovarian cancer datasets, and **(C)** qRT-PCR analysis using clinical SOC and normal control samples from Qilu Hospital of Shandong University. **(D)** ROC analysis of ALOX5AP expression for the discrimination between SOC and normal controls using TCGA-OV samples and matched normal control samples from the GTEx project. **(E)** Comparison of ALOX5AP mRNA levels across 33 TCGA cancer types and matched normal controls using GEPIA. (*p < 0.05).

Two key contemporary techniques in determining gene expression levels are RNA-Seq and microarray-based methods (38). To comprehensively compare ALOX5AP expression in ovarian cancer and normal control tissues, we cross-checked ALOX5AP mRNA levels using both of the above methods *via* searching the TNMplot database. We found that ALOX5AP was significant higher both in RNA-seq analysis, based on TCGA-OV samples and matched normal control samples from the GTEx project (fold change: 4.14, p = 5.03e-25; **Figure 1A**), and microarray analysis, based on meta-analysis of the ovarian cancer GEO data sets (fold change: 1.76, p = 4.44e-04; **Figure 1B**). To further validate these results, we used quantitative real-time PCR (qRT-PCR) assay to measure ALOX5AP expression in our clinical samples: 33 SOC and 19 normal control tissues. Concordant with the previous results, the ovarian cancer samples expressed significantly more ALOX5AP than the normal controls (fold change: 3.28, p = 0.0172; **Figure 1C**). We then performed receiver operating characteristic (ROC) analysis and found that ALOX5AP showed promising discriminating power, with an area under the ROC curve (AUC) of 0.759, suggesting that ALOX5AP may be a potential biomarker for distinguishing SOC cases from normal controls (**Figure 1D**). Last, to better understand the clinical relevance of ALOX5AP in multiple human cancers, we compared ALOX5AP mRNA levels across 33 TCGA cancer types and matched GTEx normal controls. ALOX5AP expression was significantly increased in 10/33 types and decreased in 5/33 types, demonstrating ALOX5AP have important functions in oncogenesis (**Figure 1E**). In summary, our results revealed that ALOX5AP was highly expressed in SOC, indicating that ALOX5AP may have crucial roles in ovarian carcinogenesis.

### Elevated ALOX5AP Expression Denotes Worse SOC Prognosis

**Figure 2:**
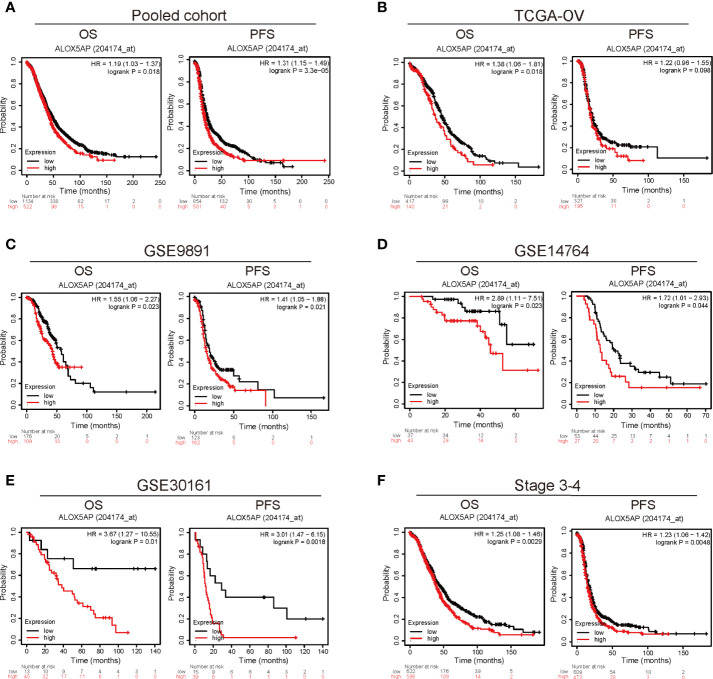
Survival analysis comparing the high and low expression of ALOX5AP in different ovarian cancer cohorts. Survival curves of OS and PFS for **(A)** pooled ovarian cancer patient cohorts, **(B)** TCGA-OV cohort, **(C)** GSE9891 datasets **(D)** GSE14764 datasets, **(E)** GSE30161 datasets. and **(F)** stage 3-4 ovarian cancer patient cohorts. (OS, overall survival; PFS, progression free survival).

To explore the prognostic role of ALOX5AP, we cross-checked survival curves based on ALOX5AP expression across multiple independent patient cohorts. We first analyzed the pooled ovarian cancer patient cohorts *via* the Kaplan–Meier plotter database: 1,656 patients with overall survival (OS) data and 1,435 patients with progression free survival (PFS) data. The results demonstrated that elevated ALOX5AP was correlated with decreased OS [HR = 1.19 (1.03–1.37), p = 0.018] and PFS [HR = 1.31 (1.15–1.49), p = 3.3e-05] (**Figure 2A**). Next, we explored the correlation between ALOX5AP expression and patient survival, using RNA sequencing data from the TCGA-OV database. Consistently, OS was significantly worse in patients with higher ALOX5AP expression [HR = 1.36 (1.06-1.81), p = 0.018]. Increased ALOX5AP was correlated to a decreased PFS, although with no statistical significance [HR = 1.22 (0.96-1.55), p = 0.098] (**Figure 2B**). To further verify the prognostic significance of ALOX5AP, we assessed another three independent ovarian cancer microarray datasets (GSE9891, GSE14764, and GSE30161). All dataset evaluations indicated that increased ALOX5AP mRNA levels were significantly associated with poor OS and PFS in ovarian cancer patients (**Figure 2C–E**). Additionally, we assessed survival rate of correlation to ALOX5AP based on different ovarian cancer stages, we found that elevated ALOX5AP expression significantly predicted worse OS and PFS in stage 3-4 patients (p < 0.05; **Figure 2F**) but not in stage 1-2 patients (p > 0.05; data not shown). We thereby concluded that elevated ALOX5AP expression was a strong predictor for worse prognosis among ovarian cancer patients.

**Figure 3:**
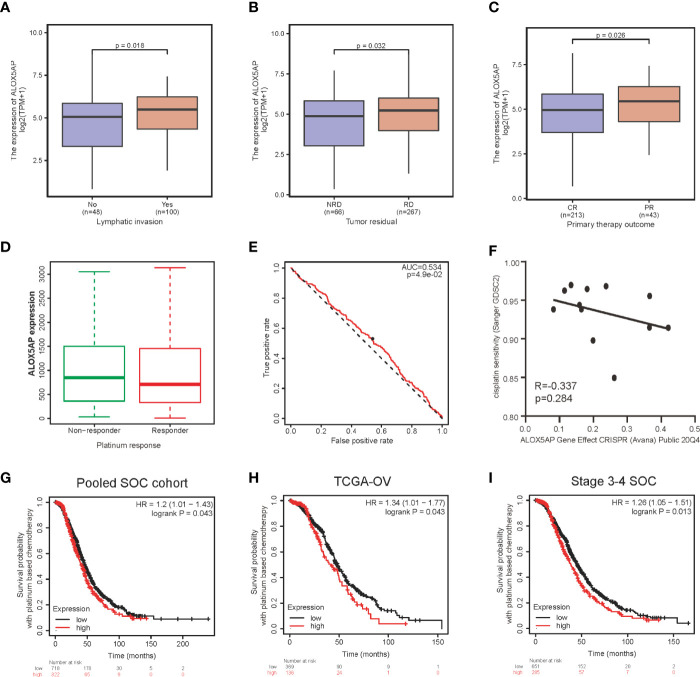
Association between ALOX5AP expression and clinicopathologic characteristics. Comparison of ALOX5AP expression in different clinical conditions including: **(A)** lymphatic invasion, **(B)** tumor residual, **(C)** primary therapy outcome and **(D)** platinum response. **(E)** Receiver operating characteristic (ROC) curve was drawn to analyze the predictive value of ALOX5AP for platinum-containing chemotherapy responsiveness. **(F)** The gene effect of ALOX5AP on cisplatin responsiveness of ovarian adenocarcinoma cell lines. Kaplan Meier curves of SOC patients receipt of platinum-based chemotherapy with high or low ALOX5AP expression in **(G)** pooled SOC patient cohorts, **(H)** TCGA-OV cohort, and **(I)** stage 3-4 SOC patient cohorts. (NRD, no residual disease; RD, residual disease; CR, complete response; PR, partial response).

Furthermore, we evaluated the association between ALOX5AP expression and clinical parameters in the TCGA-OV cohort. Consist with the above results, Wilcoxon rank sum test exhibited that higher levels of ALOX5AP were significantly correlated with more aggressive disease status, such as lymphatic invasion (p = 0.018; **Figure 3A**), residual disease (RD) after surgery (p = 0.032; **Figure 3B**), and partial response (PR) after primary therapy (p = 0.026; **Figure 3C**). Since platinum-based treatments constitute the chemotherapy standard for SOC, we then used ROC plotter to identify ALOX5AP’s effect on platinum responsiveness. The results indicated that platinum non-responders had a higher ALOX5AP expression than responders (p < 0.05; **Figure 3D**); moreover, ALOX5AP had significant predictive power for platinum responsiveness (AUC = 0.534, p = 0.049; **Figure 3E**). To further verify these results, we utilized the DepMap database to explore ALOX5AP expression and cisplatin sensitivity of 12 ovarian adenocarcinoma cell lines. Although not statistically significant, we observed a moderate negative correlation tendency (R = - 0.337, p = 0.284; **Figure 3F**). Kaplan Meier survival analysis then showed high ALOX5AP expression was correlated with poor survival rate in pooled SOC patient cohorts and TCGA-OV patients receiving platinum therapy (**Figures 3G**, **H**). Similar results were also obtained in the most aggressive stage 3-4 SOC patients, who were almost inevitably received platinum treatment (**Figure 3I**). All these results speculated that ALOX5AP levels are associated with platinum efficacy. Together, these results suggest that ALOX5AP is associated with adverse clinicopathologic characteristics and is a strong predictor of poor prognosis for SOC.

### Construction and Validation of the ALOX5AP Based Nomogram

**Figure 4:**
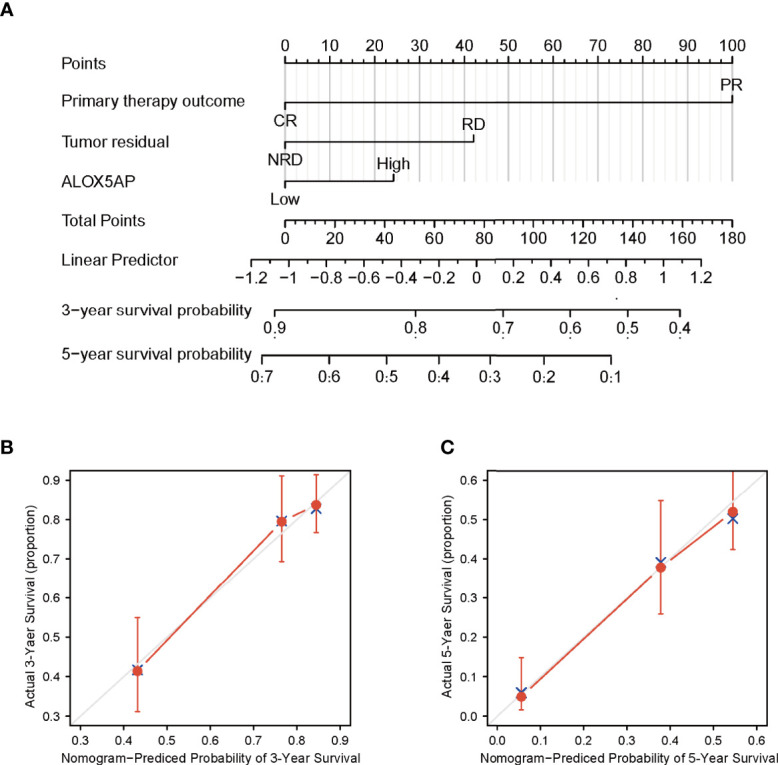
Construction and performance validation of ALOX5AP based nomogram for SOC patients. **(A)** Nomogram for predicting overall survival for ovarian cancer patients. Each predictor is assigned a score on each axis. Compute the sum of points for all predictors and denote this value as the total points. **(B, C)** Calibration curve of the nomogram for predicting 3- and 5-year OS in the TCGA-OV cohort.

We conducted univariate analysis to probe independent prognostic factors for OS *via* the Cox regression model. Clinicopathologic characteristics associated with poor OS included higher age (p = 0.017), advanced FIGO stage (p = 0.076), residual disease (RD) after surgery (p < 0.001), partial response (PR) after primary chemotherapy (p < 0.001), and notably increased ALOX5AP expression (p = 0.005). We then performed multivariate Cox analysis: ALOX5AP remained independently associated with OS (p = 0.046), along with RD (p = 0.047) and PR (p < 0.001; **Table 1**). To provide clinicians with a quantitative approach for predicting the prognosis of SOC patients, we constructed a nomogram based on the Cox multivariate analysis results, including the three above independent variables (**Figure 4A**). The survival probability of every patient could be easily estimated according to the total points of the variables. The model demonstrated good accuracy for OS prediction with a C-index of 0.683. Calibration curves revealed bias-corrected line was closed to the ideal curve (the 45-degree line), which indicated good agreement between the 3-year and 5-year survival rate estimates from the nomogram and those derived from the actual outcomes (**Figures 4B**,**C**). In sum, these findings suggested that ALOX5AP may act as an independent prognostic biomarker for SOC.

**Table 1:** Univariate and multivariate Cox proportional hazards analysis of ALOX5AP expression and OS in TCGA-OV cohort.

Characteristics	Univariate analysis		Multivariate analysis	
	HR (95% CI)	*P*	HR (95% CI)	*P*
Age (>60 vs. <=60)	1.373(1.059,1.780)	0.017*	1.350(0.982,1.855)	0.065
FIGO stage (III & IV vs. I & II)	2.085(0.925,4.699)	0.076	3.005(0.723,12.499)	0.13
Histologic grade (G3&G4 vs. G1&G2)	1.194(0.797,1.789)	0.389		
Anatomic neoplasm subdivision (Bilateral vs. Unilateral)	1.041(0.768,1.410)	0.798		
Venous invasion (Yes vs. No)	0.905(0.487,1.683)	0.753		
Lymphatic invasion (Yes vs. No)	1.422(0.839,2.411)	0.191		
TP53 status (Mut vs. WT)	0.692(0.423,1.132)	0.143		
Tumor residual (RD vs. NRD)	2.302(1.479,3.583)	<0.001*	1.709(1.006,2.904)	0.047*
Primary therapy outcome (CR vs. PR)	0.234(0.169,0.324)	<0.001*	0.285(0.199,0.407)	<0.001*
ALOX5AP (High vs. Low)	1.465(1.121,1.913)	0.005*	1.391(1.006,1.922)	0.046*

CI, confidence interval; HR, hazard ratio; RD, residual disease ;NRD, no residual disease; CR, complete response; PR, partial response.

*Statistically significant.

### Enrichment Analysis of ALOX5AP

**Figure 5:**
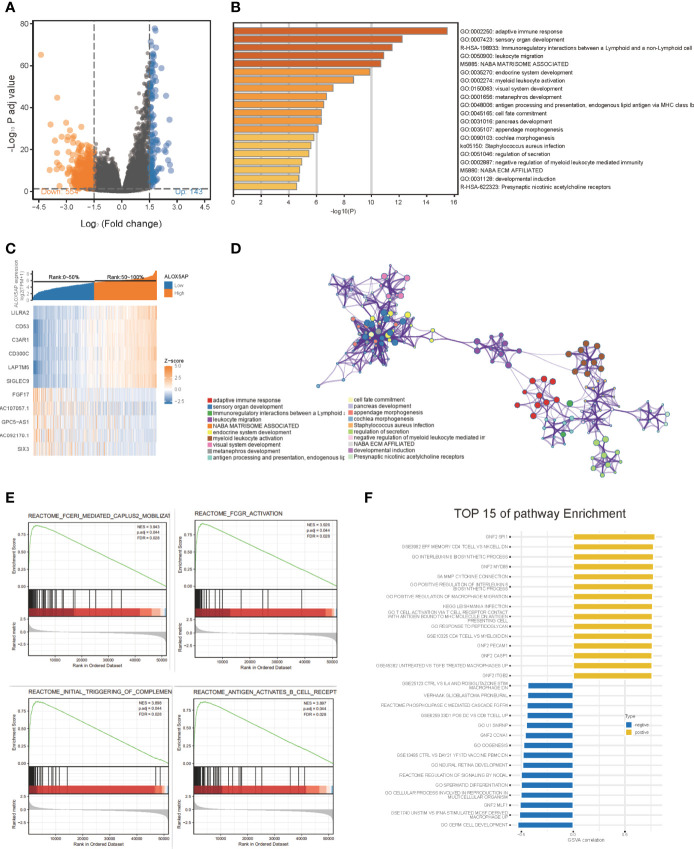
The gene enrichment analysis of ALOX5AP in SOC. **(A)** Volcano plot of the differentially expressed genes (DEGs) between ALOX5AP high and low groups in the TCGA-OV dataset. **(B)** Representative heatmap of top regulated DEGs after integrated analysis. The X-axis represents the samples, while the Y-axis denotes the differentially expressed genes. **(C)** The Bar graph demonstrating the top 20 clusters of enriched biological processes. **(D)** Visualization of the network of enriched terms colored by cluster using Metascape tool. **(E)** Enrichment plots from the gene set enrichment analysis (GSEA) in ALOX5AP high expressed samples. **(F)** Top 15 enriched signaling pathways by Gene set variation analysis (GSVA) comparison of DEGs between ALOX5AP high and low expression groups.

To gain insight into the functional role of ALOX5AP in ovarian cancer, we performed several enrichment assays. We first compared the gene expression profiles of ALOX5AP high and low groups, with the median as the cut-off value. A total of 697 differentially expressed genes (DEGs) were identified, including 143 upregulated (logFC > 1.5, p < 0.05) and 554 downregulated DEGs (logFC < -1.5, p < 0.05) (**Figure 5A**). Hierarchical clustering analysis revealed that differences in DEGs patterns could distinguish ALOX5AP high groups from low groups (**Figure 5C**). We then performed GO analysis using Metascape; the top 20 clusters are presented in **Figure 5B**, and the network of the enriched items and DEG interactions are exhibited in **Figure 5D**. Among these, adaptive immune response was the most significantly enriched function, followed by several other immune biological progresses: immunoregulatory interactions, leukocyte migration and activation, and negative regulation of myeloid leukocyte mediated immunity. In agreement with the Metascape analysis, gene set enrichment analysis (GSEA) also indicated that immune-related signaling pathways were the most enriched in the high-ALOX5AP expression group, based on their normalized enrichment score (NES; **Supplement Table 2**). The top 4 pathways were Fc epsilon receptor (FCERI) mediated Ca^2+^ mobilization, FCGR activation, initial triggering of complement, and B cell receptor (BCR) signaling activation (**Figure 5E**). Additionally, gene set variation analysis (GSVA), which provides increased power to detect subtle pathway activity changes over a sample population, further validated the above results, showing a strong correlation between ALOX5AP expression and immune regulation activities, such as positive regulation of macrophage migration and T cell activation (**Figure 5F**). Overall, our results suggest that ALOX5AP is involved in the immune regulation of tumor microenvironment of SOC.

### Correlation Between ALOX5AP and Immunocytes Infiltration

**Figure 6:**
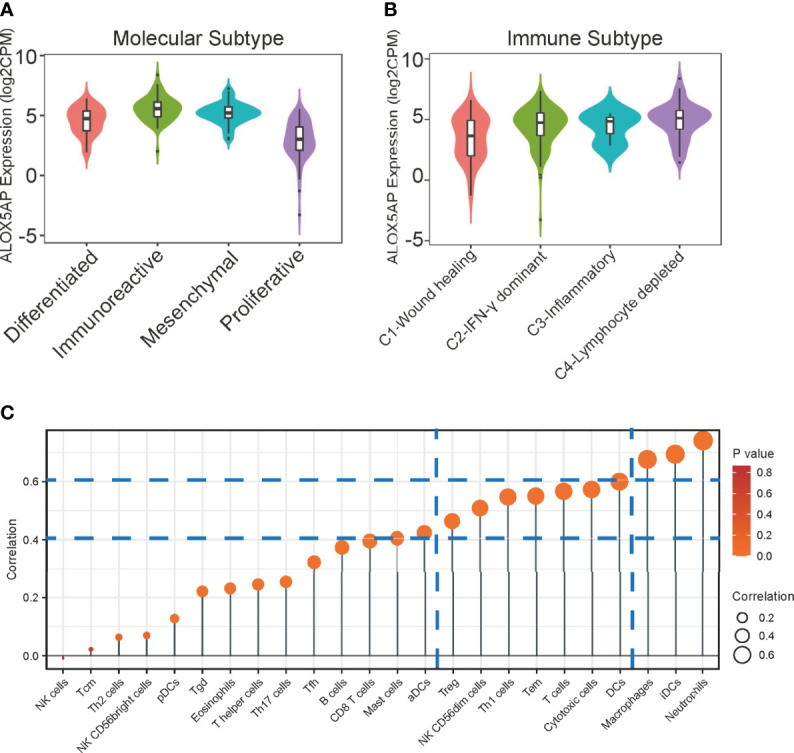
Relationship between ALOX5AP and immune infiltration. **(A)** ALOX5AP expression levels in different molecular subtypes of SOC. **(B)** ALOX5AP expression levels in different immune subtypes of SOC. **(C)** Lollipop plot shows the correlation between ALOX5AP expression and 24 immune cell subsets infiltration in SOC microenvironment. The size of dots indicates the absolute Spearman r value.

Four epithelial ovarian cancer transcriptional subtypes (differentiated, immunoreactive, mesenchymal, and proliferative) have been identified (39). When compared with other subtypes, the immunoreactive subtype exhibits the highest ALOX5AP expression (**Figure 6A**). Furthermore, when we analyzed ALOX5AP expression in different SOC immune subtypes, we found that ALOX5AP was most significantly enriched in the C4 (lymphocyte depleted) subtype (**Figure 6B**) (40). We also analyzed associations between ALOX5AP expression and immune infiltration abundance by ssGSEA. As illustrated in the lollipop plot (**Figure 6C**), the expression of ALOX5AP exhibited a strong positive correlation with infiltration abundance of inhibitory immunocytes, such as neutrophils, iDCs, and macrophages (r > 0.6, p < 0.05). Other immune cell subsets, including DCs, Cytotoxic cells, T cells, T effector memory cells, Th1 cells, NK cells, and Treg cells were moderately correlated with ALOX5AP (r > 0.4, p < 0.05). These findings suggest that ALOX5AP impact immune responses by influencing immunocytes infiltration in the ovarian cancer immune microenvironment.

### ALOX5AP and M2 Macrophage Polarization

**Figure 7:**
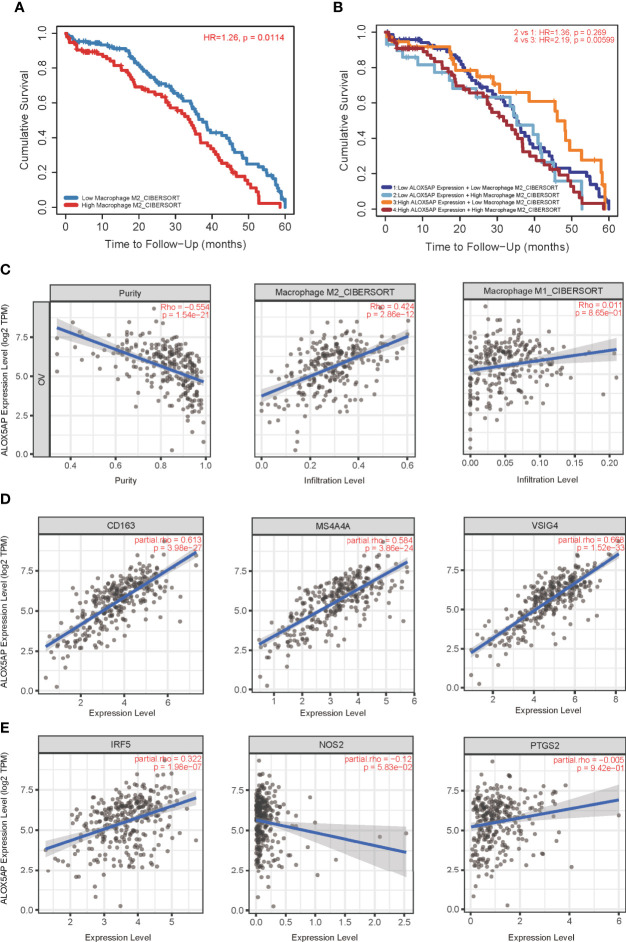
Correlation of ALOX5AP expression and M2 macrophages polarization. **(A)** Survival analysis of patients with high or low M2 macrophage infiltration in TCGA-OV cohort. **(B)** Survival time comparation in groups of different ALOX5AP expression and M2 macrophage infiltration. **(C)** Purity‐corrected Spearman’s correlation between ALOX5AP expression in ovarian cancer and two types of macrophages infiltration. The correlation between ALOX5AP and molecular biomarkers of **(D)** M2 macrophages and **(E)** M1 macrophages.

We next performed Kaplan–Meier survival analysis to evaluate the OS of ovarian cancer patients with differing immunocytes infiltration. Among the immunocytes identified above as the most related to ALOX5AP (neutrophils, iDCs and macrophages), only the high abundance of M2 macrophages was significantly associated with poor cumulative survival rate in SOC (p = 0.0114; **Figure 7A**); other lymphocytes had no significant effect on survival outcome based on the 5-year follow up analysis (data not shown). Further survival analysis revealed that patients with increased ALOX5AP expression and enriched M2 macrophage infiltration had significantly worse prognosis (**Figure 7B**). We therefore focused on the relationship between ALOX5AP and M2 macrophage infiltration. By using the state of art CIBERSORT algorithm, we found that ALOX5AP was remarkably correlated with the infiltration abundances of M2 macrophages (Spearman r = 0.424, p < 0.001), while M1 macrophages exhibited no significant correlation (r = 0.011, p = 0.865; **Figure 7C**). When we evaluated the association between ALOX5AP and biomarkers of the two macrophage types, scatter diagram revealed a highly positive correlation between ALOX5AP and M2 macrophage molecular biomarkers (CD163, MS4A4A, and VSIG4; **Figure 7D**), whereas M1 macrophage biomarkers (IRF5, NOS2, and PTGS2) showed only weak or no correlations (**Figure 7E**). Moreover, we analyzed the TISCH database with single-cell RNA sequencing data of SOC and further verified that ALOX5AP was closely related to M2 macrophage infiltration (**Supplement Figure 1**). Collectively, our findings indicate that upregulated ALOX5AP correlates with M2 macrophage polarization, which may contribute to ovarian carcinogenesis.

### Association Between ALOX5AP and Immunosuppressive Molecules

**Figure 8:**
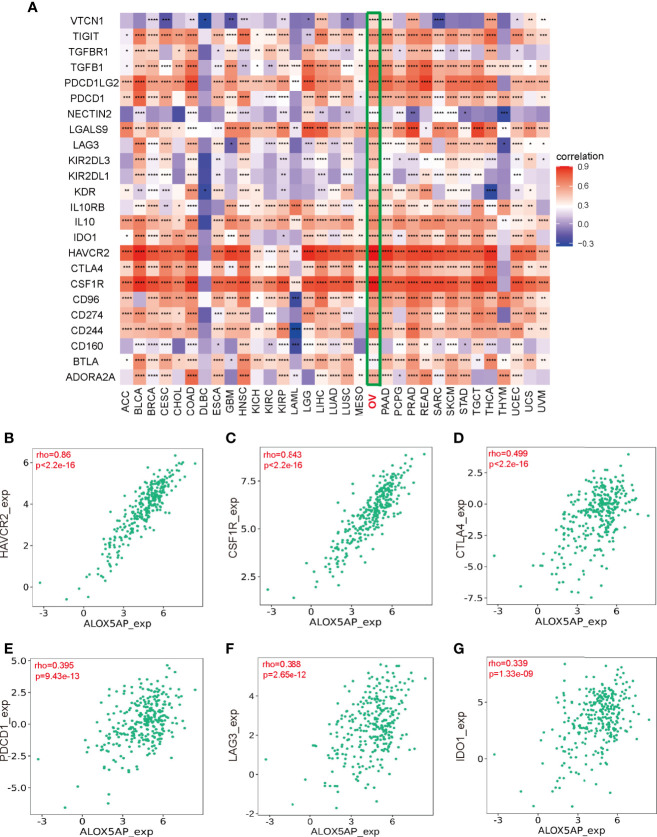
Association between Immunosuppressors and ALOX5AP expression in SOC. **(A)** Co-expression heatmap of ALOX5AP and immune inhibitory molecules across 33 TCGA tumor types. **(B–G)** Spearman correlation analysis of ALOX5AP and individual immune suppressive molecule in SOC. (*p < 0.05, **p < 0.01, ***p < 0.001, ****p < 0.0001).

To elucidate the association between ALOX5AP and immune regulation, we analyzed ALOX5AP correlation to multiple immune inhibitors across 33 TCGA cancer types (**Figure 8A**). Intriguingly, the heatmap revealed that ALOX5AP was positively correlated with an abundance of immune inhibitors in ovarian cancer, compared to other cancer types, suggesting that ALOX5AP may have a potential to serve as an immunotherapy target in SOC. We next utilized the TISIDB database to estimate the relationship between ALOX5AP and several well-known immune suppressive molecules in the ovarian cancer microenvironment. The results exhibited that ALOX5AP was strongly related to Hepatitis A virus cellular receptor 2 (HAVCR2; also known as TIM-3) and colony stimulating factor 1 receptor (CSF1R), with Spearman correlation r values > 0.8 (p < 0.001; **Figures 8B,C**). Other well-known immunosuppressors, such as cytotoxic T-lymphocyte-associated protein 4 (CTLA4), programmed cell death protein 1 (PDCD1; also known as PD1), Lymphocyte-activation gene 3 (LAG3), and indoleamine 2,3-dioxygenase 1 (IDO1) were moderately related to ALOX5AP expression (**Figures 8D–G**). Together, these results suggest that ALOX5AP may participate in mediating immunosuppression in the SOC microenvironment.

## Discussion

The tumor microenvironment (TME) for ovarian cancer is rather unique. It is primarily confined within the peritoneal cavity and frequently associated with ascites, which is rich in inflammatory cytokines and immunocytes infiltration (41). ALOX5AP was originally discovered through MK-886, which is known for its strong inhibition on the inflammatory mediator leukotriene. MK-886 exerts its function by altering the active site of ALOX5AP and thereby suppressing leukotriene (42). It is therefore probable that ALOX5AP has close relationship with inflammation and immune regulation and ultimately, the ovarian cancer microenvironment. Over the past decade, an increasing number of microarray and next-generation sequencing technologies have been used to explore novel therapeutic targets and prognostic biomarkers for various cancers, including ovarian cancer. Herein, we investigated the clinical significance of ALOX5AP in SOC using multiple bioinformatic tools as well as clinical samples. We found that ALOX5AP could be exploited as a prognostic predictor and therapeutic target *via* its effect on the ovarian cancer immune microenvironment.

Though ALOX5AP is primarily found in haematopoietic cells, aberrant expression of ALOX5AP has been detected in numerous tumor cells of non-myeloid origin, with important prognostic significance. For instance, ALOX5AP mRNA was aberrantly expressed and associated with poor prognosis in breast cancer, and inhibiting ALOX5AP activity attenuated breast cancer cell growth (43, 44). In lung cancer, Iacona et al. found that ALOX5AP was overexpressed in cancer cells compared to the normal controls and was associated with decreased OS (45). ALOX5AP has critical roles in activating 5-lipoxygenase (5-LOX), which subsequently metabolizes arachidonic acid into leukotrienes. The relationship between ovarian cancer and 5-LOX has been examined in several studies. One study found that immune stromal expression of 5-LOX was increased in ovarian cancer tissues, suggesting a role of 5-LOX signaling specifically in the tumor microenvironment during ovarian cancer development and progression (46). Another study found the transcription levels of 5-LOX and ALOX5AP in ovarian cancer cell lines were increased under hypoxic conditions (47). Although much known about the function of 5-LOX in ovarian cancer, ALOX5AP is no less important, given that ALOX5AP is critical in activating 5-LOX function. To the best of our knowledge, this is the first study to examine the effect of ALOX5AP on SOC. In this study, the results indicate that ALOX5AP is a promising biomarker for predicting unfavorable SOC prognosis.

To gain insight into the molecular mechanisms that underlie ALOX5AP mediating SOC progression, we performed functional enrichment analysis and found ALOX5AP was primarily related to immune responses and immunoregulatory interactions. The C4 (lymphocyte depleted) immune subtype of SOC had the most abundantly expressing ALOX5AP. This lymphocyte depleted subtype displayed composite signatures reflecting a macrophage dominated, low lymphocytic infiltrate, with high M2 macrophage content (40). We then evaluated the relationship between ALOX5AP expression and the infiltration of 24 immunocytes. In line with the C4 subtype, ALOX5AP expression was closely related to M2 macrophage abundance. In ovarian cancer, macrophages have been reported to facilitate tumor progression at many stages and induce immunosuppression, metastasis, and chemoresistance, subsequently leading to reduced survival (48–52). The use of the ALOX5AP inhibitor MK886, in ovarian SKOV-3 cells, has been shown to decrease macrophage migration and invasion (47). Moreover, macrophages are highly dynamic and heterogeneous, both within and across tumors, ranging from an antitumoral (so-called M1) to a pro-tumoral (so-called M2) state (53). We confirmed that ALOX5AP was closely related to M2 macrophage infiltration, evidenced by the single-cell RNA sequencing results and positively correlated M2 canonical biomarkers, while possessing an insignificant correlation with M1 markers. Previous studies have shown that M2 macrophages facilitate angiogenesis, metastasis and, most importantly, the immune suppression of ovarian cancer cells (54, 55). In line with the observations of Gao et al., we found that M2 macrophages were significantly associated with worse outcomes in SOC (56). Furthermore, our survival analysis exhibited that patients with high M2 macrophage infiltration and increased ALOX5AP expression had the worst overall outcome. Thus, poor prognosis related to elevated ALOX5AP is likely due, at least in part, to M2 macrophage polarization. Together, the present study provides preliminary evidence that ALOX5AP may participate in the induction and maintenance of M2 macrophage recruitment; translationally, targeting ALOX5AP may represent a promising approach to re-educate macrophages towards the antitumor M1 phenotype.

In addition to increasing immunosuppressive cell abundance, tumors escape immune surveillance (i.e. immune evasion) by expressing immune checkpoint inhibitory molecules (57). Previous studies have found that the 5-LOX/ALOX5AP pathway plays important roles in manipulating the TME by affecting cancer-related immune evasion (58). In this study, compared to other cancer types, in ovarian cancer most of the immunosuppressive hallmarks were significantly positively associated with ALOX5AP expression. These results are in agreement with the C4 lymphocyte depleted immune subtype, which indicates a suppressed immune microenvironment. Notably, our study found that crucial immune repressive receptors—which have gained significant attention in ovarian cancer research, such as TIM3, CSF1R, CTLA4, PD1, and LAG3 as well as inhibitory enzymes such as IDO1—were closely related to ALOX5AP expression. Among them, TIM3, which had the highest Spearman correlation r value with ALOX5AP, has been reported to negatively regulate antitumor immunity by inducing active T cell exhaustion and exerting an antiproliferative effect in the ovarian cancer TME (59, 60). For CSF1R, several studies have exhibited that its blockade significantly decreases ascites and M2 macrophage infiltration in epithelial ovarian cancer mouse models (61, 62). CTLA4, a negative regulator of T-cell activation, was approved as the first immune checkpoint in melanoma treatment and anti-CTLA4 immunotherapy has shown outstanding antitumor efficacy in clinical trials of ovarian cancer patients (63). One of the most notable biomarkers in cancer research PD1, binds to its ligand PD-L1 and negatively regulates T cell activation, resulting in immune evasion; moreover, M2 macrophages’ ability to block tumor-specific T cell activity is thought to be *via* their influence on PD1 (64). PD1/PDL1 axis inhibitors have been investigated as single-agent therapies in ovarian cancer treatment (65). LAG3, which is expressed by activated T-cellws has been reported to act on ovarian cancer infiltrating lymphocytes (TILs) and dampen antitumor immunity in collaboration with PD1 (66). Similarly, IDO1—an enzyme with an immune tolerance effect—has been reported to regulate peritoneal dissemination and is linked to poorer survival in ovarian cancer patients (67). All these molecules deemed to reduce antitumor immune responses were found in this study to be closely associated with ALOX5AP expression at a genomic level. The results strongly implicate ALOX5AP as a possible co-contributor to immune evasion in the ovarian cancer immune microenvironment. Accumulating evidence suggests that several immune checkpoints coordinate against immune attacks and that the targeting of single immune checkpoint may have limited efficacy. Our results suggest that ALOX5AP provides a synergistic effect with multiple immunosuppressive molecules; thereby, playing a special role in immune evasion. ALOX5AP may serve as a useful adjunct target in future immune checkpoint inhibitor ovarian cancer treatment.

Indeed, several ALOX5AP inhibitors have been developed and are currently under clinical investigation as treatments for respiratory and cardiovascular diseases, such as the classical prototype MK-886 and the follow-up MK-0591, Bay-X1005, and DG-031 (first licensed by DeCode Genetics and then developed by Bayer) (68). Notably, ALOX5AP inhibitors have superior effects in females because androgens impede the leukotriene biosynthetic 5-LOX/ALOX5AP complex assembly (69). This sex bias suggests that ovarian cancer patients may benefit from superior efficacy and reduced side effects when treated with ALOX5AP inhibitors. Given the availability of well-studied, highly effective ALOX5AP inhibitors, our current work is of paramount translational significance. One exciting but reasonable speculation is that combining immune-checkpoint blockade and ALOX5AP inhibitor therapies could synergistically halt ovarian cancer progression by simultaneously combating M2 macrophage infiltration and immune suppression in the ovarian cancer microenvironment. Future clinical trials are needed to evaluate efficacy before ALOX5AP becomes a widely accepted prognostic indicator and therapeutic target of SOC.

Although this study improved our understanding of the oncogenic role of ALOX5AP in ovarian cancer progression and development, there were several limitations. First, all ovarian cancer patient cohorts utilized were retrospective. A major limitation was the retrospective design which may cause information bias. The clinical value of ALOX5AP needs to be further validated in prospectively designed studies, which may decrease the bias caused by uncontrollable factors. Second, though the genomic investigation has become one of the most efficient method of accelerating translational cancer research, the correlation analysis based on bioinformatic algorithms can provide preliminary evidence of the relationship rather than determine the causal relationship between ALOX5AP and immune activities regulation. Due to the complexity of immunoregulation in cancer pathogenesis and progression, the exact molecular mechanisms of ALOX5AP in mediating immune response remain to be fully elucidated. Further explorations of the potential direct or indirect interplay between ALOX5AP and immune activities were warranted. Nevertheless, the current study provides foundation evidence supporting ALOX5AP as an immune related promising prognostic biomarker for SOC.

In conclusion, ALOX5AP is upregulated in SOC and is associated with worse clinicopathologic characteristics and consequently, a poorer prognosis. ALOX5AP participates in SOC progression *via* M2 macrophage recruitment and polarization as well as mediating immune suppression in the tumor immune microenvironment. This study offers promising insights into the prognostic and predictive biomarker roles of ALOX5AP and may provide a novel approach for tailored immunotherapy in the fight against SOC.

## Data Availability Statement

The datasets analyzed for this study can be found in the TCGA (https://portal.gdc.cancer.gov/) and Gene Expression Omnibus (https://www.ncbi.nlm.nih.gov/geo/).

## Ethics Statement

The studies involving human participants were reviewed and approved by Ethical Committee of Shandong University. The patients/participants provided their written informed consent to participate in this study.

## Author Contributions

XY and MG conceived and designed the study. XY, LA, XW, CZ, WH, CS, RL, HM, and HW collected and analyzed the data. XY, LA, XW, CZ, WH, and CS wrote the original draft. RL, HW, HM, and MG reviewed and edited the manuscript. All authors contributed to the article and approved the submitted version.

## Funding

This work was supported by National Natural Science Foundation of China (81502245) and Shandong Provincial Natural Science Foundation (ZR2014HQ016).

## Conflict of Interest

The authors declare that the research was conducted in the absence of any commercial or financial relationships that could be construed as a potential conflict of interest.

## Supplementary Material

Supplementary Figure 1 Single-cell RNA sequencing analysis of ALOX5AP expression and immunocytes infiltration using TISCH database. UMAP plot of all the single cells, with each color coded for **(A)** 3 major cell types, **(B)** 5 minor cell types, and **(C)** ALOX5AP expressed cells.
