# Supplementary figures and images for: ALOX5AP Predicts Poor Prognosis by Enhancing M2 Macrophages Polarization and Immunosuppression in Serous Ovarian Cancer Microenvironment
Source: Front Oncol. 2021 May 19;11:675104. doi: 10.3389/fonc.2021.675104 (PMC8172172; doi:10.3389/fonc.2021.675104)

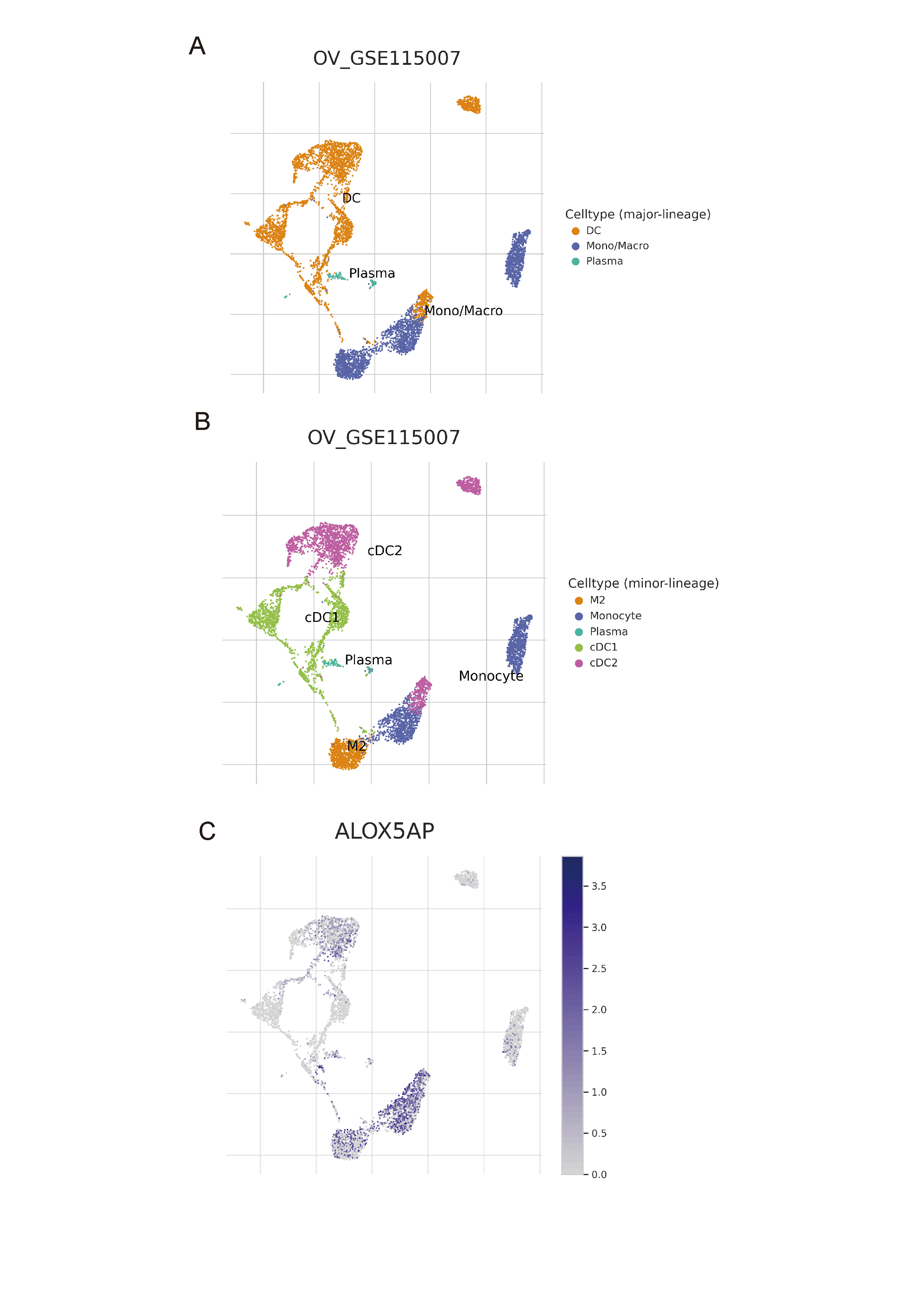

Supplement: Supplementary Figure 1 — Single-cell RNA sequencing analysis of ALOX5AP expression and immunocytes infiltration using TISCH database. UMAP plot of all the single cells, with each color coded for (A) 3 major cell types, (B) 5 minor cell types, and (C) ALOX5AP expressed cells. [file Image_1.tif]
