# Supplementary material for: ALOX5AP Predicts Poor Prognosis by Enhancing M2 Macrophages Polarization and Immunosuppression in Serous Ovarian Cancer Microenvironment
Source: Front Oncol. 2021 May 19;11:675104. doi: 10.3389/fonc.2021.675104 (PMC8172172; doi:10.3389/fonc.2021.675104)
Supplement: Supplementary file 2 [file Table_1.pdf]

Supplement table 1： Clinical characteristics of patients with ovarian cancer

Characters	Cases, n	Percentage (%)
FIGO stage		
Stage I	1	0.3
Stage II	22	5.9
Stage III	293	78.6
Stage IV	57	15.3
Histologic grade		
G1	1	0.3
G2	42	11.5
G3	322	88.0
G4	1	0.3
Race		
White	326	90.1
Black or African American	25	6.9
Asian	11	3.0
Anatomic neoplasm subdivision		
Bilateral	253	71.5
Unilateral	101	28.5
Venous invasion		
No	40	38.8
Yes	63	61.2
Lymphatic invasion		
No	48	32.4
Yes	100	67.6
TP53 status		
Mut	248	90.5
WT	26	9.5
Tumor residual		
NRD	66	19.8
RD	267	80.2
Primary therapy outcome		
CR	213	69.8
PR	43	14.1
SD	22	7.2
PD	27	8.9
