# Supplementary material for: ALOX5AP Predicts Poor Prognosis by Enhancing M2 Macrophages Polarization and Immunosuppression in Serous Ovarian Cancer Microenvironment
Source: Front Oncol. 2021 May 19;11:675104. doi: 10.3389/fonc.2021.675104 (PMC8172172; doi:10.3389/fonc.2021.675104)
Supplement: Supplementary file 3 [file Table_2.pdf]

Supplement Table 2

Gene sets enriched in the samples of SOC in the high expression of ALOX5AP group					
ID	SetSize	Enrichment	NES	p value	FDR
REACTOME_FCERI_MEDIATED_CAPLUS2_MOBILIZATION	84	0.883	3.943	0.012	0.028
REACTOME_FCGR_ACTIVATION	67	0.918	3.926	0.011	0.028
REACTOME_INITIAL_TRIGGERING_OF_COMPLEMENT	77	0.888	3.898	0.011	0.028
REACTOME_ANTIGEN_ACTIVATES_B_CELL_RECEPTOR_BCR_LEADING_TO_GENERATION	83	0.868	3.897	0.011	0.028
REACTOME_FCERI_MEDIATED_MAPK_ACTIVATION	85	0.877	3.894	0.012	0.028
REACTOME_CD22_MEDIATED_BCR_REGULATION	59	0.919	3.873	0.009	0.028
REACTOME_CREATION_OF_C4_AND_C2_ACTIVATORS	69	0.898	3.847	0.011	0.028
REACTOME_ROLE_OF_LAT2_NTAL_LAB_ON_CALCIUM_MOBILIZATION	69	0.894	3.833	0.011	0.028
REACTOME_ROLE_OF_PHOSPHOLIPIDS_IN_PHAGOCYTOSIS	78	0.869	3.815	0.011	0.028
REACTOME_SCAVENGING_OF_HEME_FROM_PLASMA	66	0.876	3.748	0.011	0.028
KEGG_LEISHMANIA_INFECTION	70	0.831	3.586	0.011	0.028
KEGG_HEMATOPOIETIC_CELL_LINEAGE	84	0.801	3.576	0.012	0.028
KEGG_GRAFT_VERSUS_HOST_DISEASE	37	0.934	3.526	0.006	0.028
KEGG_ANTIGEN_PROCESSING_AND_PRESENTATION	81	0.799	3.526	0.012	0.028
PID_IL12_2PATHWAY	61	0.828	3.522	0.009	0.028
REACTOME_COSTIMULATION_BY_THE_CD28_FAMILY	69	0.815	3.491	0.011	0.028
REACTOME_CHEMOKINE_RECEPTORS_BIND_CHEMOKINES	46	0.859	3.428	0.007	0.028
KEGG_ALLOGRAFT_REJECTION	35	0.922	3.407	0.006	0.028
KEGG_INTESTINAL_IMMUNE_NETWORK_FOR_IGA_PRODUCTION	45	0.849	3.377	0.007	0.028
REACTOME_INTERLEUKIN_10_SIGNALING	41	0.873	3.35	0.007	0.028
PID_TCR_PATHWAY	63	0.789	3.335	0.009	0.028
REACTOME_INTERFERON_GAMMA_SIGNALING	90	0.759	3.328	0.014	0.031
KEGG_AUTOIMMUNE_THYROID_DISEASE	50	0.818	3.315	0.008	0.028
REACTOME_GENERATION_OF_SECOND_MESSENGER_MOLECULES	32	0.91	3.281	0.005	0.028
PID_IL12_STAT4_PATHWAY	33	0.88	3.253	0.006	0.028
PID_CD8_TCR_DOWNSTREAM_PATHWAY	65	0.743	3.22	0.01	0.028
KEGG_TYPE_I_DIABETES_MELLITUS	41	0.837	3.21	0.007	0.028
PID_CD8_TCR_PATHWAY	52	0.789	3.206	0.009	0.028
REACTOME_INTERLEUKIN_2_FAMILY_SIGNALING	44	0.808	3.191	0.007	0.028
PID_NFAT_TFPATHWAY	44	0.801	3.164	0.007	0.028
REACTOME_PD_1_SIGNALING	23	0.959	3.109	0.005	0.028
KEGG_ASTHMA	28	0.9	3.1	0.005	0.028
REACTOME_DAP12_INTERACTIONS	39	0.812	3.085	0.007	0.028
PID_FCER1_PATHWAY	59	0.731	3.084	0.009	0.028
PID_IL23_PATHWAY	37	0.817	3.083	0.006	0.028
REACTOME_PHOSPHORYLATION_OF_CD3_AND_TCR_ZETA_CHAINS	22	0.949	3.078	0.005	0.028
REACTOME_ANTIGEN_PROCESSING_CROSS_PRESENTATION	96	0.684	3.063	0.014	0.031
PID_BCR_5PATHWAY	63	0.719	3.04	0.009	0.028
REACTOME_TRANSLOCATION_OF_ZAP_70_TO_IMMUNOLOGICAL_SYNAPSE	19	0.977	3.029	0.005	0.028
KEGG_PRIMARY_IMMUNODEFICIENCY	35	0.813	3.006	0.006	0.028
KEGG_B_CELL_RECEPTOR_SIGNALING_PATHWAY	75	0.692	3.006	0.011	0.028
REACTOME_TNFS_BIND_THEIR_PHYSIOLOGICAL_RECEPTORS	27	0.878	3.003	0.005	0.028
